# Concordance of FDG PET/CT metabolic tumour volume versus DW-MRI functional tumour volume with T2-weighted anatomical tumour volume in cervical cancer

**DOI:** 10.1186/s12885-017-3800-9

**Published:** 2017-12-06

**Authors:** Alta Y. T. Lai, Jose A. U. Perucho, Xiaopei Xu, Edward S. Hui, Elaine Y. P. Lee

**Affiliations:** 1 Department of Radiology, Pamela Youde Nethersole Eastern Hospital, Chai Wan, Hong Kong Special Administrative Region, China; 2 Department of Diagnostic Radiology, Queen Mary Hospital, Li Ka Shing Faculty of Medicine, The University of Hong Kong, Room 406, Block K, 102 Pokfulam Road, High West, Hong Kong Special Administrative Region, China

**Keywords:** Uterine cervical neoplasms, Positron-emission tomography, Fluorodeoxyglucose F18, Radiation oncology, Image-guided radiotherapy, Intensity-modulated radiotherapy

## Abstract

**Background:**

^18^F–fluoro-deoxyglucose positron emission tomography with computed tomography (FDG PET/CT) has been employed to define radiotherapy targets using a threshold based on the standardised uptake value (SUV), and has been described for use in cervical cancer. The aim of this study was to evaluate the concordance between the metabolic tumour volume (MTV) measured on FDG PET/CT and the anatomical tumour volume (ATV) measured on T2-weighted magnetic resonance imaging (T2W-MRI); and compared with the functional tumour volume (FTV) measured on diffusion-weighted MRI (DW-MRI) in cervical cancer, taking the T2W-ATV as gold standard.

**Methods:**

Consecutive newly diagnosed cervical cancer patients who underwent FDG PET/CT and DW-MRI were retrospectively reviewed from June 2013 to July 2017.

Volumes of interest was inserted to the focal hypermetabolic activity corresponding to the cervical tumour on FDG PET/CT with automated tumour contouring and manual adjustment, based on SUV 20%–80% thresholds of the maximum SUV (SUVmax) to define the MTV_20–80_, with intervals of 5%.

Tumour areas were manually delineated on T2W-MRI and multiplied by slice thickness to calculate the ATV.

FTV were derived by manually delineating tumour area on ADC map, multiplied by the slice thickness to determine the FTV_(manual)_. Diffusion restricted areas was extracted from *b*0 and ADC map using K-means clustering to determine the FTV_(semi-automated)_.

The ATVs, FTVs and the MTVs at different thresholds were compared using the mean and correlated using Pearson’s product-moment correlation.

**Results:**

Twenty-nine patients were evaluated (median age 52 years). Paired difference of mean between ATV and MTV was the closest and not statistically significant at MTV_30_ (−2.9cm^3^, −5.2%, *p* = 0.301). This was less than the differences between ATV and FTV_(semi-automated)_ (25.0cm^3^, 45.1%, *p* < 0.001) and FTV_(manual)_ (11.2cm^3^, 20.1%, *p* = 0.001). The correlation of MTV_30_ with ATV was excellent (*r* = 0.968, *p* < 0.001) and better than that of the FTVs.

**Conclusions:**

Our study demonstrated that MTV_30_ was the only parameter investigated with no statistically significant difference with ATV, had the least absolute difference from ATV, and showed excellent positive correlation with ATV, suggesting its superiority as a functional imaging modality when compared with DW-MRI and supporting its use as a surrogate for ATV for radiotherapy tumour contouring.

## Background

Precise determination of cervical tumour boundary is important in radiotherapy to deliver the highest possible radiation dose to cancerous tissues while minimizing that to surrounding healthy tissues.

Given its superior soft tissue contrast, MRI is the modality of choice for the anatomical delineation of tumour outline and local tumour extent, especially in determining whether parametrial invasion is present to differentiate early from advanced stage disease. Despite excellent spatial resolution, delineation of tumour extent can be limited using conventional T2-weighted (T2W) sequences in certain scenarios, e.g. isointense tumours and diffusely infiltrative lesions, in assessing response of tumours to therapy and, in particular, in differentiating residual or recurrent disease from post-treatment fibrosis due to the overlap of morphological appearances [1].

The clinical utilisation of functional imaging in gynaecological malignancy is evolving [2–4]. In the era of more sophisticated treatment options such as image-guided adaptive radiotherapy, functional imaging techniques such as diffusion-weighted MRI (DW-MRI) and ^18^F–fluoro-deoxyglucose positron emission tomography integrated with computed tomography (FDG PET/CT) have been demonstrated to provide information for more precise definition of radiation target [5–7]. DW-MRI allows characterization of biological tissues based on their water diffusion property that changes with the integrity of cellular membranes and tissue cellularity [8]. Quantitative assessment can be derived from the apparent coefficient diffusion (ADC) maps obtained from DW-MRI [9]. For instance, ADC has been used to differentiate between normal and cancerous cervical tissue, and the latter was found to correlate negatively with tumour cellular density and grading [10, 11]. Additional DW-MRI has been demonstrated in the literature to outperform T2W imaging alone in depicting local recurrence and differentiating it from post-treatment changes such as fibrosis [12–14]. The use of ADC for measuring target volumes with different tissue characteristics for dose prescription in image-guided adaptive brachytherapy [15] and various segmentation methods with DW-MRI [16] have also been studied. However, further investigation in its clinical application to radiotherapy treatment planning is warranted.

FDG PET/CT utilises FDG, a glucose analogue, to provide valuable metabolic information based on the increased glucose uptake and glycolysis of cancer cells, and can depict metabolic abnormalities before morphological alterations occur [17]. FDG PET/CT has been employed to define radiotherapy targets using a threshold based on the standardised uptake value (SUV) for over a decade [18], and that for cervical cancer has been recently demonstrated [19]. Modification of radiation treatment volumes to FDG-avid lymph nodes and primary tumour can facilitate the accurate definition of tissues with metabolically active disease and the avoidance of normal tissue; hence allowing dose boosts to FDG-avid tumour volumes and lower doses to the bone marrow, urinary bladder and rectum [20, 21].

Despite promising results of using functional imaging to delineate radiation target in cervical cancer, the segmentation methods and thresholds used are highly variable in the literature. The aim of this study is to evaluate the concordance between the metabolic tumour volume (MTV) measured on FDG PET/CT and the anatomical tumour volume (ATV) measured on T2W imaging; and compared with the functional tumour volume (FTV) measured on DW-MRI in cervical cancer, using the T2W ATV as gold standard [22].

## Methods

### Patient selection

The retrospective study was reviewed by local institutional review board and informed consent was waived. We reviewed the local database and all consecutive patients with newly diagnosed cervical cancers who underwent both FDG PET/CT and MRI as pre-treatment imaging from June 2013 to July 2017 were included. Cases with incomplete inclusion of the tumour on MRI were excluded. The median time difference between the two examinations was 4 days (range 0 to 32).

### FDG PET/CT

#### Patient preparation and image acquisition

Whole-body FDG PET/CT (coverage from the skull base to the upper one third of the thighs) was performed on a combined PET/CT scanner (Discovery VCT, 64 multislice spiral CT; GE Healthcare Bio-Sciences Corp.), using a standardised protocol. After 6 h of fasting, 222–370 MBq (4.8 MBq/kg) of weight-adjusted FDG was administered intravenously. Following a 60-min uptake time, whole-body emission PET was obtained with 6 bed positions of 2 min and 30 s acquisition time in each bed position. PET was attenuated with CT data and reconstructed with an ordered-subset expectation maximization iterative reconstruction algorithm (14 subsets and 2 iterations) and subsequently fused with CT images for further analysis. The CT imaging parameters were as follows: 120 kVp; 200–400 mA; 0.5 s per CT rotation; pitch, 0.984:1; and 2.5-mm intervals, with or without 60–100 mL (1.5 mL/kg) intravenous contrast medium.

#### Metabolic tumour volume (MTV)

**Fig. 1:**
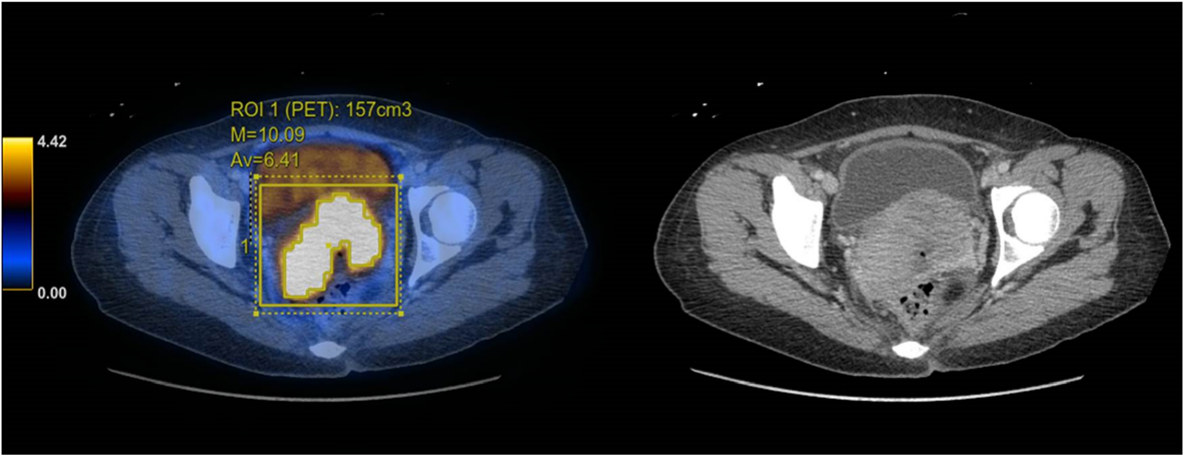
MTV was calculated by the thresholding method on FDG PET/CT. Focal activity in the uterine cervix was identified. A VOI was inserted manually, carefully avoiding the urinary bladder. SUVmax was quantified by the software automatically. The tumour was outlined as the region encompassed by a given fixed percent intensity level relative to the maximum activity in the tumour. 20% to 80% thresholds of the SUVmax (MTV_20_ to MTV_80_) at intervals of 5% were used in this study. MTV: metabolic tumour volume; VOI: volume of interest; SUVmax: maximum standardized uptake value

Both SUV and volumetric analysis were performed using Advantage Volume Share on ADW 4.7 workstation (GE Healthcare, Chicago, Illinois, United States). Focal hypermetabolic activity in the uterine cervix corresponding to the cervical tumour was visually identified, where a 3D volume of interest (VOI) was inserted (Fig. 1). Automated tumour contouring with manual adjustment was performed to include the boundaries of the lesion in the axial, coronal, and sagittal planes, and to avoid the urinary bladder. SUV measurement was performed by normalization of the injected dose to lean body mass. Lean body mass was used for normalization instead of total body mass because it is less dependent on body habitus across populations [23]. Maximum SUV (SUVmax) was automatically generated. MTV was measured using an SUV-based automated contouring program. The voxels presenting SUV ≥ 20% to 80% thresholds of the SUVmax within the contouring margin were incorporated to define the metabolic tumour volumes (MTV_20_ to MTV_80_), with intervals of 5%.

### MRI

#### Patient preparation and image acquisition

Patients were prepared for MRI after 6 h of fasting and 20 mg hyoscine butylbromide (Buscopan, Boehringer Ingelheim, Germany) was given intramuscularly at the start of each examination to reduce bowel peristalsis. All examinations were performed on a 3.0-T MRI system (Achieva 3.0 T TX, Philips Healthcare, Best, the Netherlands) using a dedicated 16-channel phased array torso coil.

The standard sequences included sagittal T2 W turbo spin-echo (TSE) and an oblique axial T2W TSE (perpendicular to the long axis of the cervix). Additional axial T2W TSE was acquired to ensure the same anatomical coverage and slice profile as the DW-MRI. Post-contrast 3D T1 W TSE was acquired after DW-MRI (Table 1).

**Table 1:** Summary of MRI scan parameters

*Sequences*	*T2-Weighted TSE*	*T2-Weighted SPAIR*	*T2-Weighted TSE*	*T2-Weighted TSE*	*DWI*	*CE 3D T1-weighted TSE*
Plane	Sagittal	Coronal	Axial	Oblique Axial	Axial	3D
TR/TE (ms)	4000/80	3500/80	2800/100	2800/100	2000/54	3/1.4
Turbo factor	30	21	12	14	NA	NA
Field of view (mm)	240 × 240	230 × 230	402 × 300	220 × 220	406 × 300	370 × 203
Matrix size	480 × 298	352 × 300	787 × 600	316 × 311	168 × 124	248 × 134
Slice thickness (mm)	4	4	4	4	4	1.5
Intersection gap (mm)	0	0	0	0	0	0
Bandwidth (Hz/pixel)	230	186	169	162	15.3	724
Number of excitations	2	1	1	1	2	1

CE: contrast-enhanced, DWI: diffusion-weighted imaging; TR/TE: repetition time/echo; TSE: turbo spin echo

DW-MRI was performed using single-shot spin-echo echo-planar imaging, immediately after the axial T2W TSE imaging. It was acquired in free breathing with background body signal suppression (presaturation inversion recovery fat suppression) and parallel imaging with sensitivity encoding [SENSE] factor of 2 (Table 1). Image acquisition with 13 *b*-values (0–1000 s/mm^2^) were performed in the axial plane covering 20 slices to include the entire cervical cancer, using motion-probing gradients in three orthogonal axes to generate the geometric averaged DW signal. The full inclusion of the entirety of the tumour on the DW-MRI images was confirmed visually for every case.

#### Anatomical tumour volume (ATV)

Tumour areas were manually delineated on T2W images in sagittal and oblique axial planes and multiplied by the slice thickness to calculate the sagittal and oblique axial tumour volumes. Two reviewers, EL (8-year experience in MRI with special interest in gynaecological oncology imaging) and AL (5-year experience in MRI), separately placed the ROIs on the T2W images in the sagittal and oblique axial planes, respectively. The volumes were averaged between the two reviewers to determine the ATV.

#### Functional tumour volume (FTV)

**Fig. 2:**
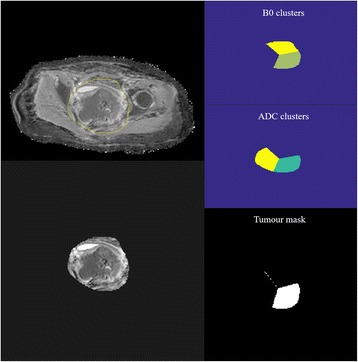
A semi-automated method was used to extract diffusion restricted areas from corresponding *b*0 and ADC map. VOIs were manually inserted to include the entire tumour. Voxels were automatically separated into 3 groups based on ADC values using a K-means clustering method: solid tumour mass with high cellularity having intermediate ADC, fat and fibrotic tissues having low ADC and normal tissue with low cellularity or cystic tissues having high ADC. FTV_(semi-automated)_ was hence calculated by discarding the fat and fibrotic cluster and the normal tissue cluster, leaving the solid tumour mass cluster. ADC: apparent diffusion coefficient; FTV: functional tumour volume

Averaged DW signal was used to generate the ADC maps using the Levenberg-Marquardt fitting algorithm under the mono-exponential model described by the function:

$$ \frac{S_b}{S_0}=\left[{e}^{-b\bullet ADC}\right] $$

where S_b_ represents the mean signal intensity with the diffusion gradient, *b*, S_0_ is the mean signal intensity when *b* = 0 s/mm^2^. VOIs were manually drawn by two reviewers, EL and AL, for each lesion. The first set of VOIs were strict manual delineations of the tumour by both reviewers and excluded the surrounding normal tissue based on the hypointense signal of the tumour on the ADC map with cross reference to the axial T2W images. FTV by the two reviewers was then calculated using these VOIs multiplied by slice thickness. The volumes were averaged between the two reviewers to determine the FTV_(manual)_. The second set of VOIs was drawn by the same two reviewers, EL and AL, to include all of the tumour and did not require exclusion of surrounding normal tissue. Volumetric k-means clustering was then used to automatically separate voxels in the tumour volume into three groups based on S_0_ and ADC values. These groups were defined as: solid tumour mass with high cellularity having intermediate ADC and intermediate S_0_ intensities; normal tissue with low cellularity or cystic tissues having high ADC [5, 24] and high S_0_ intensities; fat and fibrotic tissues having low ADC low S_0_ intensities. A study by *Gong* et al. [25] has shown that slice-by-slice K-means clustering, using both S_0_ images and ADC, is a promising method for reliable delineation of heterogeneous tumours in patients with metastatic gastrointestinal stromal tumours. FTV_(semi-automated)_ was calculated by discarding the fat and fibrotic cluster and the normal tissue cluster, leaving the solid tumour mass cluster. Parametric map generation and semi-automatic functional volume segmentation were performed using in-house programs using MATLAB (The Mathworks Inc., Natick, MA, USA) (Fig. 2). The volumes were averaged between the two reviewers to determine the FTV_(semi-automated)_.

### Statistical analysis

The ATVs measured on T2W images, FTVs on DW-MRI and the MTVs at different thresholds on FDG PET/CT were compared. The ATVs, FTVs and MTVs were correlated using Pearson’s product-moment correlation. R version 3.4.1 (R Foundation for Statistical Computing, Vienna, Austria) was used for statistical analysis. A two-tailed *p*-value < 0.05 was considered statistically significant.

## Results

### Demographics

A total of 29 patients were evaluated with median age of 52 years (range 27–76 years). Further clinicopathological characteristics were tabulated in Table 2.

**Table 2:** Patient demographics including age, clinical tumour staging and histological types

Median age in years (range)		52 (27–76)
		No of patients, *n* (%)
FIGO stage	IB	8 (27.6%)
	IIA	2 (6.9%)
	IIB	7 (24.1%)
	IIIB	10 (34.5%)
	IVB	1 (3.4%)
	Unstaged	1 (3.4%)
Histology	Squamous cell carcinoma	16 (55.2%)
	Adenocarcinoma	8 (27.6%)
	Others	5 (17.2%)

### Quantitative measurements

Mean SUVmax of the cervical tumours was 9.2, range 3.3–16.7. Mean ADC of the cervical tumours was 0.934 +/− 0.120 mm^2^/s. The mean ATVs measured in sagittal and oblique axial planes are 51.7 and 59.3 cm^3^ respectively.

### Paired differences of mean between (ATV and FTV) vs (ATV and MTV at different SUV thresholds)

The paired difference of mean between ATV and MTV_30_, mathematically represented by mean ATV – mean MTV_30_, was −2.9 cm^3^, −5.2%, *p* = 0.301. This difference was not statistically significant and was the closest to ATV compared with all other FTVs and MTVs measured at other SUVmax thresholds, including the differences between ATV and FTV_(semi-automated)_ (mean ATV – mean FTV_(semi-automated)_ = 25.1 cm^3^, 45.1%, *p* < 0.001) and between ATV and FTV_(manual)_ (mean ATV – mean FTV_(manual)_ = 11.2 cm^3^, 20.1%, *p* = 0.001). The means of the ATV, FTV_(semi-automated)_, FTV_(manual)_ and MTV_20_ to MTV_80_ (with intervals of 5%) and the differences of their means with ATV are shown in Table 3.

**Table 3:** Means of the ATV, FTV_(semi-automated)_, FTV_(manual)_ and MTV_20_ to MTV_80_

	Mean (cm^3^)	Paired difference with mean ATV (cm^3^)	(%)	*p* value
ATV	55.5			
FTV_(manual)_	44.3	11.2	20.1%	0.001
FTV_(semi-automated)_	30.5	25.1	45.1%	<0.001
PET MTV_20_	83.3	−27.8	−50.0%	<0.001
PET MTV_25_	69.2	−13.7	−24.7%	0.001
**PET MTV**_**30**_	58.4	−2.9	−5.2%	**0.301**
PET MTV_35_	49.0	6.5	11.8%	0.026
PET MTV_40_	41.4	14.1	25.3%	0.001
PET MTV_45_	34.9	20.6	37.1%	<0.001
PET MTV_50_	29.3	26.2	47.2%	<0.001
PET MTV_55_	24.3	31.2	56.2%	<0.001
PET MTV_60_	19.4	36.1	65.0%	<0.001
PET MTV_65_	15.0	40.5	73.0%	<0.001
PET MTV_70_	11.0	44.5	80.2%	<0.001
PET MTV_75_	7.3	48.2	86.9%	<0.001
PET MTV_80_	4.4	51.1	92.1%	<0.001

The differences of their means with ATV. The paired difference of mean between ATV and MTV_30_ was not statistically significant and was the closest to ATV compared with all other FTVs and MTVs (BOLD)

### Correlation

The correlations of MTV_20–50_ with ATV were excellent (*r* > 0.9, *p* < 0.001), and those of MTV_30–40_ were better than that with FTV_(semi-automated)_ and FTV_(manual)_. There was a gradual decline in correlation of MTVs with ATV as the percentage threshold of SUVmax increased (Table 4).

**Table 4:** Correlation of MTVs at all SUVmax thresholds were significantly correlated with T2W anatomical volume and with DW-MRI functional volume (*p <* 0.05)

	Pearson’s product-moment correlation (r)	*p* value
ATV	1.000	
FTV_(manual)_	**0.963**	<0.001
FTV_(semi-automated)_	**0.956**	<0.001
PET MTV_20_	**0.955**	<0.001
PET MTV_25_	**0.962**	<0.001
PET MTV_30_	**0.968**	<0.001
PET MTV_35_	**0.975**	<0.001
PET MTV_40_	**0.968**	<0.001
PET MTV_45_	**0.948**	<0.001
PET MTV_50_	**0.921**	<0.001
PET MTV_55_	0.877	<0.001
PET MTV_60_	0.830	<0.001
PET MTV_65_	0.803	<0.001
PET MTV_70_	0.777	<0.001
PET MTV_75_	0.777	<0.001
PET MTV_80_	0.781	<0.001

Pearson correlation coefficient (r) larger than 0.9 are highlighted in **bold**. ATV: anatomical tumour volume; FTV: functional tumour volume; MTV: metabolic tumour volume

## Discussion

Our study demonstrated that among all metabolic threshold levels and FTV_(semi-automated)_ and FTV_(manual)_, the MTV_30_ had the least absolute difference from ATV and was the only parameter investigated which did not show a statistically significant difference from ATV. In addition, MTV_30_ showed excellent positive correlation with ATV.

MRI is indispensable in the local disease assessment of cervical cancer. The ability of combined functional volume assessment and local disease extent using MRI alone could present as a promising imaging algorithm for patients with cervical cancer. However, the evidence to support this is limited in the current literature and most studies have used the manual segmentation method based on DW-MRI images [26, 27].

The choice of imaging modality used in tumour contouring or segmentation technique can result in varying derived tumour volume [16, 28, 29]. There is no consensus of the methodology of tumour segmentation using DW-MRI or ADC values. Clustering is a method, which groups similar data, and the k-means algorithm is often chosen for image segmentation and grouping voxels of same signal intensities and has been used for classification of functional imaging data [30]. This algorithm is simple and efficient and has been shown to be able to differentiate ADC values of benign and malignant pathologies [24]. A recent study has shown that K-means clustering using both S_0_ and ADC is a promising method for reliable delineation of heterogeneous tumours in patients with metastatic gastrointestinal stromal tumours [25]. The relative signal intensity [31] and region growing [32] methods are alternative segmentation techniques which were described to have limitations related to their dependence on *b*-value and acquisition method for DW-MRI images, and sensitivity to signal-to-noise ratio [16].

FDG PET/CT has the advantage of identifying the metabolic activity and providing information on tumour biology. It is increasingly recognized as a useful tool for directing RT planning during intensity-modulated radiation therapy, volumetric-modulated arc treatment [20] and image-guided brachytherapy [33], thereby allowing targeted dose escalation to target tissues with high metabolic activity, and reducing dose to surrounding tissues [34]. The utility of FDG PET/CT has been shown to lead to less gastrointestinal toxicity in patients with gynaecological malignancies [35].

Various tumour segmentation techniques using FDG PET/CT exist: manual contouring, which consists of visual assessment for determining tumour outline; thresholding, which uses a minimum SUV value to identify target; and gradient edge detection, in which tumour delineation is based on the changes in signal across a given area [36]. As SUV thresholding has been the focus in initial investigative approaches and is the most commonly employed method of FDG/PET-based tumour volume segmentation [18], it was the segmentation technique of choice in this study.

Volume concordance between FDG PET/CT, and T2W and DW-MRI imaging in cervical cancer has been previously observed [26, 27, 37], and tumour sub-volumes with increased metabolic activity on FDG PET/CT was found to have greater cell density by DW-MRI [38]. Zhang et al. suggested that PET-measured gross tumour volume using an SUVmax threshold method may increase the accuracy in target volume delineation when performed on a sequential FDG PET/MRI platform [37]. SUV-based primary squamous histology cervical tumour volume estimation at 30% to 35% of SUVmax values correlated significantly with volume on MRI [27]. In a hybrid FDG PET/MRI study, volume measurement using 35% or 40% thresholds of the SUVmax has been found to display a strong concordance with the tumour volumes measured on T2W and DW-MRI in cervical cancer [27, 37].

In our study, the mean of differences between ATV and MTV was the smallest with MTV_30_, concordant with previous literature [27]. Although DW-MRI also gives information on tumour cellularity and the semi-automated method may potentially reduce processing time and inter-observer variability, our study suggested that the FTVs, regardless of the segmentation methods, borne larger differences from the ATV than MTV_30_ did. Moreover, contouring based on FDG PET/CT can be performed in a semi-automated fashion and this feature is readily available on standard workstation, which is easy to use, providing the merit of reducing the time required for processing, and potentially also improving inter-observer agreement, as shown previously by studies on tumour delineation for rectal and lung cancers [39–41].

Furthermore, MTV_30_, having the least absolute difference from ATV, being the only parameter investigated with no statistically significant difference from ATV, and having an excellent positive correlation with ATV supported its use as a surrogate for ATV for radiotherapy tumour contouring and dose escalation; with the benefit of having metabolic information available for characterizing the biological features of the tumour and optimizing the use of individualized, conformal and biologically effective radiation therapy.

## Conclusion

In conclusion, MTV delineation on FDG PET/CT appears promising and superior as a functional imaging modality when compared with DW-MRI in tumour contouring with MTV_30_ being the best correlate to ATV.

## Abbreviations

ADC: apparent diffusion coefficient
ATV: anatomical tumour volume
CE: contrast-enhanced
DW-MRI: diffusion weighted magnetic resonance imaging
FDG PET/CT: ^18^F–fluoro-deoxyglucose positron emission tomography with computed tomography
FTV: functional tumour volume
MRI: magnetic resonance imaging
MTV: metabolic tumour volume
PET: positron emission tomography
SUV: standardised uptake value
SUVmax: maximum standardised uptake value
T2W: T2-weighted
TR/TE: repetition time/echo
TSE: turbo spin echo
VOI: 3D volume of interest
